# Effects of a Single Intravitreal Injection of Aflibercept and Ranibizumab on Glomeruli of Monkeys

**DOI:** 10.1371/journal.pone.0113701

**Published:** 2014-11-21

**Authors:** Alexander Tschulakow, Sarah Christner, Sylvie Julien, Maximilian Ludinsky, Markus van der Giet, Ulrich Schraermeyer

**Affiliations:** 1 Section of Experimental Vitreoretinal Surgery, Centre for Ophthalmology, Tuebingen, Germany; 2 STZ OcuTox Preclinical Drug Assessment, Hechingen, Germany; 3 Division of Nephrology, Charité University Medicine, Charité Campus Benjamin Franklin, Berlin, Germany; University of Houston, United States of America

## Abstract

**Purpose:**

It is known that endothelial cells in the kidney are also strongly VEGF-dependent. Whether intravitreal drugs can be detected within the glomeruli or affect VEGF in glomerular podocytes is not known. Therefore, the aim of this pilot study was to investigate the effects of a single intravitreal injection of aflibercept and ranibizumab on glomeruli of monkeys.

**Methods:**

The kidneys of eight cynomolgus monkeys, which were intravitreally injected either with 2 mg of aflibercept or with 0.5 mg of ranibizumab, were investigated one and seven days after injection. Two animals served as controls. The distribution of aflibercept, ranibizumab and VEGF was evaluated using anti-Fc- or anti-F(ab)-fragment and anti-VEGF antibodies respectively. The ratio of stained area/nuclei was calculated using a semi-quantitative computer assisted method. Glomerular endothelial cell fenestration was quantified in electron microscopy using a systematic uniform random sampling protocol and estimating the ratio of fenestrae per µm.

**Results:**

Compared to the controls, the anti-VEGF stained area/nuclei ratio of the ranibizumab-treated animals showed no significant changes whereas the stained areas of the aflibercept-treated monkeys showed a significant decrease post-treatment. Immune reactivity (IR) against aflibercept or ranibizumab was detected in aflibercept- or ranibizumab treated animals respectively. The number of fenestrations of the glomerular endothelial cells has shown no significant differences except one day after aflibercept injection in which the number was increased.

**Conclusion:**

Surprisingly, both drugs could be detected within the capillaries of the glomeruli. After a single intravitreal injection of aflibercept, VEGF IR in the podocytes was significantly reduced compared to controls. Ranibizumab injection had no significant effect on the glomeruli's VEGF level. Whether this is caused by aflibercept's higher affinity to VEGF or because it is used in a higher stoichiometric concentration compared to ranibizumab remains to be investigated.

## Introduction

Vascular endothelial growth factor (VEGF) is a 43- to 46-kd glycoprotein and a major regulator of physiological and pathological angiogenesis [1]. It increases vascular permeability and plays a vital role in endothelial cell migration, proliferation and survival. In the kidney, VEGF is highly expressed in presumptive as well as in mature podocytes and plays a critical role in glomerular development and function i.e. to establish the glomerular filtration barrier [2].

Anti-VEGF-agents were first used in cancer treatment with some severe side effects in consequence of systemic administration. Concerning the kidneys, proteinuria and hypertension have been reported [3]–[5]. In addition, thrombotic microangiopathy, nephrotic syndrome, bowel perforation, haemorrhages, stroke, myocardial infarction, decreased pulmonary surfactant and delayed wound healing may occur [6]–[9].

Also in ophthalmology, excessive angiogenesis is a pathogenic factor in many diseases. These include diabetic proliferative retinopathy and age-related macular degeneration (AMD) in adults and retinopathy of prematurity in infants. In the pathogenesis of wet AMD, VEGF plays an outstanding role as it appears to be sufficient and essential in both physiological and pathological angiogenesis [10], [11]. Bevacizumab (Avastin, Genentech/Roche), used in an off-label manner in ophthalmology, is a full length antibody, as such carries the Fc-fragment and is therefore kept in circulation by the binding to the neonatal Fc receptor (FcR) [12]. The importance of the FcR for pharmacokinetics of agents containing the Fc domain was also shown in animal models [13]. Besides good clinical results in ophthalmologic treatment, adverse effects like arterial thromboembolic events, hypertension and renal thrombotic microangiopathy were observed [14]–[17]. Our group has extensively described the effects of intravitreally injected bevacizumab on monkey eyes [18]–[21]. Local ocular effects like reductions in choriocapillaris fenestrations, alteration of choroidal blood flow [19], formation of immune complexes and thrombotic microangiopathy [20], [21] have been reported.

Ranibizumab (Lucentis, Genentech/Novartis) was approved in 2006 by the food and drug administration (FDA) for the treatment of wet AMD after the first off-label uses of bevacizumab. As a cleavage product of bevacizumab, it only consists of a Fab fragment and similarly to bevacizumab it blocks the receptor binding domain of all isoforms of VEGF-A. In contrast to the latter, its modified molecular structure aims to avoid immunological reactions. Aflibercept (VEGF Trap-Eye/Eylea, Regeneron/Bayer) is the latest FDA approved agent for ophthalmic use. It possesses binding sequences for VEGFR-1 and VEGFR-2 that were fused to the Fc segment of human IgG1 antibody with a binding affinity that was 140 times greater than that of ranibizumab and binds to all VEGF-A isoforms, VEGF-B and PIGF [22].

After intravitreal injection of anti-angiogenic agents, Csaky et al., detected these substances in the systemic circulation [16] and there is evidence that anti-VEGF drugs reach the systemic circulation sufficiently to decrease serum VEGF concentrations [10], [23], [24]. Heiduschka et al. have shown in monkeys that some 2% to 5% of bevacizumab is already in the blood stream one and four days after intravitreal injection [18]. Bevacizumab with its long serum half-life of 20 days can lower blood VEGF levels even when intravitreally administrated in an amount comparable to that achieved with intravenous therapy [10], [25], [26]. This finding raises the possibility that intravitreally administered bevacizumab may suppress baseline physiologic VEGF activity. Ranibizumab, in contrast, has a much shorter serum half-life of 6 hours and its serum levels remain low. Although renal complications have been reported in only rare cases after intravitreal injections of ranibizumab or bevacizumab [14], [17], [27]–[29], the possibility of systemic complications after intravitreal injections of anti-VEGF agents has to be considered. Regarding aflibercept, little data concerning systemic effects after intravitreal injection is available although a clearance from the eye into the systemic circulation has been shown [30]. Therefore, it is vital to investigate the effects of anti-VEGF agents in nonhuman primates to establish biologic activity and adverse effects relevant to humans. This *in vivo* study was performed in monkeys as antibody molecules and their interaction with Fc receptors in monkeys mimic those present in humans [31]. To this aim, we investigated the effects of a single intravitreal injection of ranibizumab and aflibercept on the kidneys of eight monkeys on days one and seven after injection. The distribution of aflibercept and ranibizumab in renal glomeruli was analysed, VEGF levels in glomeruli and the glomerular endothelial cell fenestrations were quantified.

Note that due to the high costs of experiments, this pilot study only consists of a small number of animals and the study design does not allow the evaluation of a great variety of time points.

## Materials and Methods

### 1. Animals and study protocol

Ten cynomolgus monkeys (Macaca fascicularis, aged 3 to 8 years) were raised at the Covance Laboratories (Muenster, Germany) under standard conditions. All animals were housed and handled in strict accordance with good animal practice under supervision of veterinarians in accordance with the German Animal Welfare Act and were monitored for evidence of disease and changes in attitude, appetite or behaviour suggestive of illness. Full details are shown in Table 1.

**Table 1:** Details of the animals used in this study.

Covance Study 8260977	Covance Study 8274007
Species: Cynomolgus monkeys (*Macaca fasicularis)*	Sprecies: Cynomolgus monkey (*Macaca fasicularis)*
Origin: Mauritius	Origin: Mauritius
Age at predose start: 3 to 8 years	Age at predose start: 3 to 8 years
Predose body weight: 4 to 12 kg	Predose body weight: 4 to 12 kg
Number and sex: 5 healthy male animals	Number and sex: 5 healthy male animals
Animal housing: Pair and single, due to the single control animal	Animal housing: Pair and single, due to the single control animal

The naive Cynomolgus fascicularis monkeys are from a closed breeding colony (Noveprim Mauritius). Tests for TB, B-Virus, SIV, SRV, STLV, are carried out during export and import quarantine. Additionally tests for TB, B-Virus, SIV, SRV, STLV are carried out regularly for all animals on site regardless if they are in studies or not.

Cynomogus monkeys are housed in social groups before and during studies. The space requirements are according to the EU directive (DIRECTIVE 2010/63/EU OF THE EUROPEAN PARLIAMENT AND OF THE COUNCIL of 22 September 2010 on the protection of animals used for scientific purposes).

The animal live under a 12 hour dark light cycle, have ad libidum access to water and food provided twice daily, lab diet plus fresh fruit. The foremost enrichment is social housing. Additionally mirrors, wooden trunks, balls are supplied as a standard. Further enrichment devices are available and made available on a rotating scheme.

The animal welfare officer on site regularly checks the housing and handling conditions.

Since animals are housed in groups during the study the individuals are not randomised to the dose group, but rather a stable group of animals created a long time before the study.

Both eyes of four animals were intravitreally injected with ranibizumab and another four animals with aflibercept. The doses of anti-VEGF agents were the same as clinically used in humans and as provided and recommended by the manufacturers: 2 mg of aflibercept and 0,5 mg of ranibizumab, respectively.

All injections were carried out in the morning, following slight sedation with ketamin and diazepam. One and seven days after intravitreal injection, the animals were sacrificed under general anaesthesia and the kidneys were removed (except the left kidney of a monkey that was sacrificed on day seven after injection of aflibercept showing aplasia). The left kidney of each animal was prepared for immunohistochemistry, specimens of the right kidneys served for electron microscopy as described below. One untreated monkey sacrificed on day seven and one monkey injected with aflibercept's vehicle and sacrificed one day after injection served as controls. Blood samples were taken before injection (predose) and on days one and seven after injection. Platelet-poor plasma was prepared by centrifugation. For injection specifications, ophthalmic examinations as well as fixation methods and histological procedures, please see our previous publications [21], [32].

### Ethical Statement

Handling and housing of the animals were only done at Covance Laboratories GmbH. All animals were housed and handled in strict accordance with good animal practice under supervision of veterinarians in accordance with the German Animal Welfare Act and were monitored for evidence of disease and changes in attitude, appetite or behaviour suggestive of illness. The animals were sacrificed, and their kidneys were fixed at Covance Laboratories GmbH. The animals were sacrificed under general anaesthesia, i.e., intramuscular injection of ketamine hydrochloride followed by an intravenous sodium pentobarbitone (Lethabarb, Virbac, Australia) overdose. Only the further investigations: electron microscopy and immunohistochemistry were performed in our lab in Tuebingen. These investigations did not necessitate approval by an institutional review board. Covance Laboratories GmbH test facility is fully accredited by the AAALAC. This study was approved by the local IACUC, headed by Dr. Jörg Luft, and performed in consideration of the following recommendation:

Commission Recommendation 2007/526/EC on guidelines for the accommodation and care of animals used for experimental and other scientific purposes (Appendix A of Convention ETS 123).

### 2. Kidneys samples and fixation

On days one and seven after intravitreal injection, the animals were sacrificed under general anaesthesia, i.e., intramuscular injection of ketamine hydrochloride followed by an intravenous sodium pentobarbitone (Lethabarb, Virbac, Australia) overdose. The kidneys were extracted five minutes post-mortem. The left kidney of each animal was fixed in paraformaldehyde uncut for immunohistochemistry, a specimen of the right kidneys was dissected into small cube-like pieces with a length of 2–3 mm and then fixed in glutaraldehyde (ice cooled) for electron microscopy (the only available kidney of the monkey described above was prepared for electron microscopy). The kidneys of the monkey without treatment, or with aflibercept's vehicle injection, were handled in the same manner.

### 3. Immunohistochemistry

Sections (4 µm) were cut from formalin-fixed, paraffin embedded tissue and mounted on superfrost Plus microscope slides (R. Langenbrinck Labor- u. Medizintechnik).

The slides were deparaffinised and rehydrated, and heat induced epitope retrieval in TRIS EDTA (pH 9) using a pressure cooker was performed. After two washing steps in TBS (0.5% TWEEN) immunohistochemical staining of VEGF was performed according to the instructions provided by the manufacturer in a humid chamber. The slides were incubated for 60 min with the primary mouse anti-VEGF antibody (1∶50, DAKO, Denmark) at 37°C and then processed using the DAKO REAL Detection System Alkaline Phosphatase/RED kit rabbit/mouse, then counterstained with hematoxylin and covered. The same procedure was performed for immune reactivity analysis against ranibizumab and aflibercept using respectively a primary mouse antibody against the human IgG-Fab-fragment (Dianova, 1∶100) and a primary mouse antibody against the human IgG-Fc-fragment (Dianova, 1/150). These samples were used for the quantification and normalisation of VEGF or ranibizumab/aflibercept stainings.

Additionally an immune reactivity analysis using fluorescent antibodies was performed. A mouse antibody against the human IgG-Fab-fragment of IgG (dilution 1∶400, Jackson ImmunoResearch) and a goat anti-mouse alexa488 labelled secondary antibody (dilution 1∶400, Invitrogen) were used for ranibizumab staining. Goat anti-human IgG-Fc antibody (dilution 1∶200, Novus Biologicals) and a donkey anti-goat alexa488 labelled secondary antibody (dilution 1∶500, molecular probes) were used for aflibercept staining.

### 4. Quantification and normalisation of VEGF and ranibizumab/aflibercept stainings

**Figure 1:**
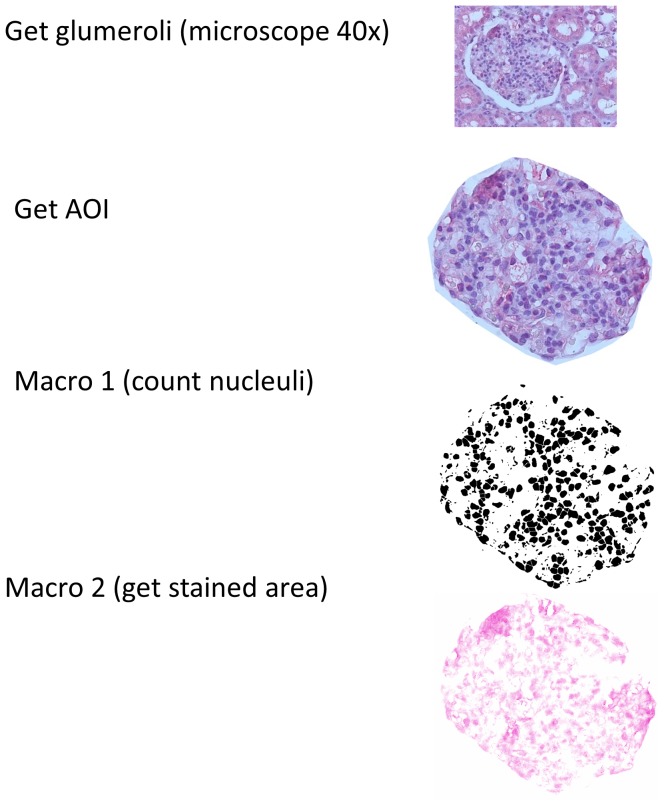
Semi-quantitative computer assisted method used for the quantification and normalisation of the VEGF staining. Glomeruli were defined as the area of interest (AOI), and then the AOI were isolated using the image j software. The nuclei in the AOI were then counted and finally the stained area of each AOI was determined.

From each tissue sample, photos from 10 randomly chosen glomeruli-containing regions were taken using a Zeiss Axioscop microscope with AxioVision image capture software at a magnification of 400x. On these photos the glomeruli were defined as the area of interest (aoi). These aoi were isolated using the image j software. In the next step the nuclei in the aoi were counted using a semi-quantitative computer assisted method (image j). Then the stained area of each aoi was determined (Fig. 1). Briefly, the image j program offers a colour deconvolution plug which splits images into three color-channels and is widely used for immunohistochemistry analysis [33]. The number of nuclei could be calculated after the hematoxilin stained areas were isolated using the “H&E” filter of the color deconvolution plug in. After that “watershed” and “particle counter” algorithms were run for the nuclei count as described by others [34], [35]. This method was compared with manual counting and was found to be as accurate as the manual method. For the determination of the stained area we used the colour deconvolution plug in with the RGB (red, green, blue) filter. The red image was used for the calculation of the stained area.

For the determination of the background for the stained area determination the red dye intensity from a control which was treated only with the secondary antibody and the red dye was set as threshold for the filter settings for the image j analysis. The ratio of stained area/nuclei was calculated and the statistical analysis was performed as further described.

### 5. VEGF-A plasma levels

Blood samples of all monkeys were collected in tubes containing EDTA before intravitreal injection (predose) and on day one and seven after injection of anti-VEGF agents, and plasma was prepared by centrifugation, transferred to new tubes and stored at −70 C. Plasma samples were analyzed using commercially available ELISA kits for human VEGF-A (DVE00) (R&D Systems, Minneapolis, Minnesota). Briefly, the microtitration plates were coated with monoclonal antibodies specific for VEGF-A, standards and probes were added, incubated and washed. Then, an enzyme-linked polyclonal antibody specific for VEGF-A was added and its substrate solution followed after a second incubation and wash step. After stopping the colour development, the intensity of colour (Optical Density) was measured by photospectrometry with the lower detection limit of VEGF-A set at 30 pg/ml. Calculation of VEGF concentration was performed according to the manufacturer's recommendations.

### 6. Light and electron microscopy

Specimens were postfixed with 1% OsO4 at room temperature in 0.1 M cacodylate buffer (pH 7.4), en bloc stained with uranyl acetate and lead citrate, and embedded in Epon after dehydration in a graded series of acetones. Semithin sections were stained with Toluidine Blue and examined by light microscopy (Zeiss Axioplan2 imaging, Zeiss, Jena, Germany). For electron microscopy, the sections were cut ultrathin and analyzed with a Zeiss 902 A electron microscope (Zeiss, Jena, Germany).

### 7. Quantification of the glomerular endothelial fenestrations

**Figure 2:**
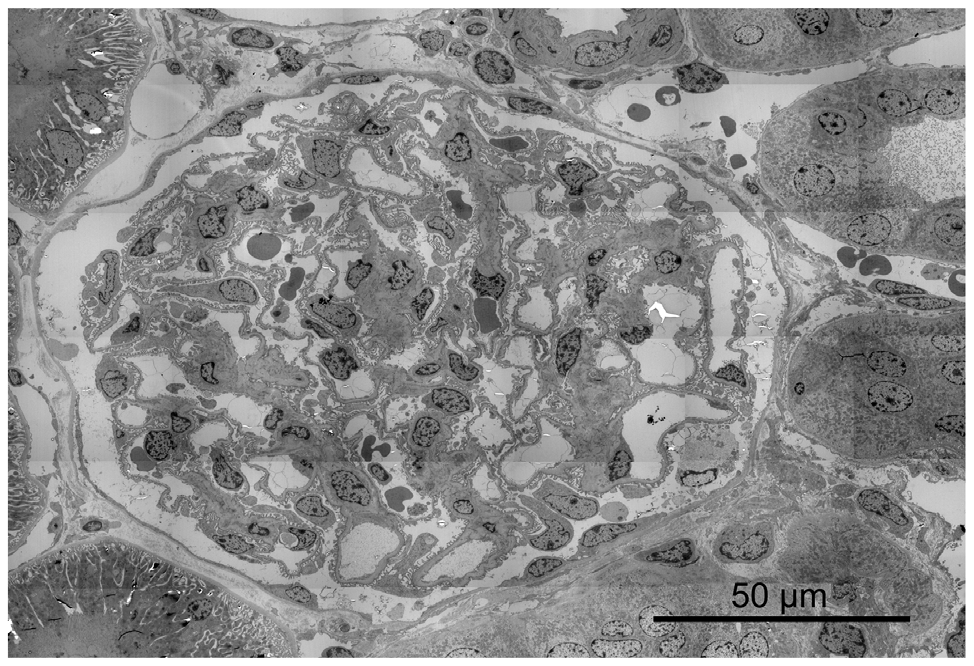
Example of a montage of transmission electron micrographs by using multiple image arrangements (MIAs) in order to provide the entire renal glomerulus. These images were taken using the image analysis software iTEM at the magnification of ×3000. Here one day after aflibercept injection.

**Figure 3:**
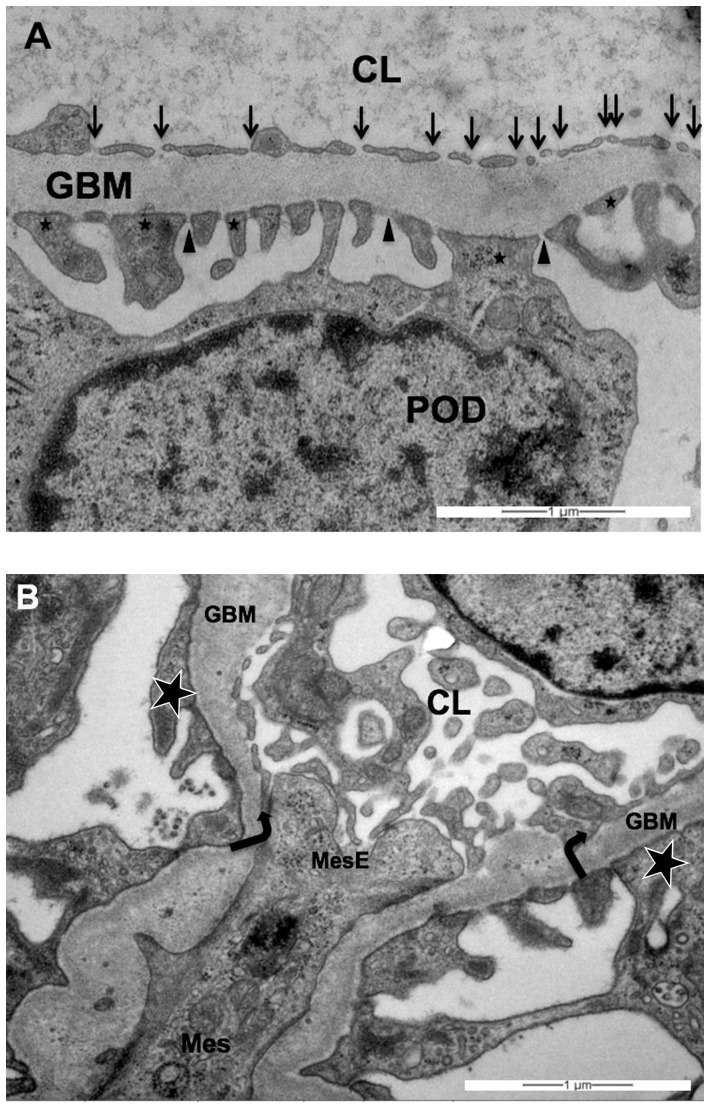
Examples of representative transmission electron micrographs of (A) a fenestrated glomerular endothelium and of (B) peripheral *versus* mesangial portions of the glomerular endothelium (both one day after aflibercept injection). (**A**) Blood lumen on the upper part, urinary space on the lower part of the image. The healthy glomerular filtration barrier consists of three layers [6]: the fenestrated glomerular endothelial cells, the intervening glomerular basement membrane and the podocyte processes and slit diaphragm. GBM =  glomerular basement membrane, CL =  capillary lumen, POD =  podocyte. Arrows mark glomerular endothelial cells fenestrae (note the absence of diaphragm), asterisks mark podocyte foot processes, arrowheads mark podocyte slit diaphragm. (**B**) At this magnification, podocyte foot processes (asterisks) allow the clear identification of the capillary lumen (CL). In accordance with our definition, the peripheral portion begins where the endothelium and the glomerular endothelial basement membrane (GBM) run approximately parallel (marked by arrows). Arrows mark direction into which peripheral endothelium begins. In between the arrows the mesangium (Mes) and the mesangial portion of the capillary endothelium (MesE) is located. Note that in the mesangial portion there is no GBM adjacent to the fenestrated endothelium so that the described counting method is not applicable and the endothelium does not show the typical single- layered configuration. Magnification ×20000.

Under the light microscope at a low magnification (x10) glomeruli were identified in semi-thin sections and checked on mechanical or fixation artifacts and pathological features at a higher magnification. Three glomeruli per kidney (six per time point for ranibizumab and aflibercept, three for controls) with middle to large diameter and intact bowman's capsules were chosen and cut ultrathin for electron microscopy. After examination of the probes at a magnification of 3000 fold, montages of transmission electron micrographs were performed by using the multiple image arrangements  =  MIAs in order to provide montages of the entire glomeruli (Fig. 2). The montages consist of overlapping images that were taken using an image analysis software (iTEM, Olympus Soft Imaging Solutions, Muenster, Germany). Similarly to a previously described method [36], high-magnification images were taken from the chosen glomeruli according to a systematic uniform random sampling protocol (SURS). Starting at the top-most portion of the glomerular tuft, ×20000 images were taken moving the position of the thin section grid with the help of the X and Y grid position control keys. For this purpose, the grid was moved ten units horizontally taking a picture at each stop point until the opposite portion of the capsule was reached. Then the position of the grid was moved 10 units vertically and 5 units to the right or left, respectively. This procedure was continued until the entire glomerular profile was scanned through (Fig. S1). After pictures were scanned through for artefacts by a blinded observer, high- magnification images were used for quantification of glomerular fenestration using a counting tool of the iTEM software. Only pictures on which glomerular endothelium was undoubtedly identifiable were analyzed. A line was drawn and measured along the lamina rara interna of the endothelial basement membrane adjacent to the fenestrated endothelium and fenestrations were counted by a single observer under standardised conditions. As capillary walls can be divided into peripheral and mesangial regions [36], only peripheral portions where glomerular basement membranes and capillary walls show parallelism were considered. We found that the quantification in the mesangial portions is not comparable to the peripheral parts as the predominant amount of mesangial endothelium is not fenestrated whereas on the other hand there are parts with increased fenestration (as described as “alveolus fenestratus endothelialis” by Kondo et al., [37]. Thus, this endothelium is morphologically not comparable to the single layered endothelium in the peripheral portions so that applying the same quantification method would not be admissible (Fig.3A–B). Therefore, the mesangial portions were excluded from the quantification of the fenestrations.

### 8. Statistical analysis

The ratio of fenestrae per µm was calculated using Microsoft-Office-Excel for each image considered. Statistical significances for the evaluation of the fenestrae per µm of the glomerular capillaries and for the quantification of VEGF as well as ranibizumab/aflibercept were determined by using the Student's t test from the JMP10 statistical program (SAS, Heidelberg, Germany). P<0.05 was considered statistically significant.

## Results

### 1. Immunohistochemistry

#### **1.1. Ranibizumab/aflibercept fluorescence staining.**

**Figure 4:**
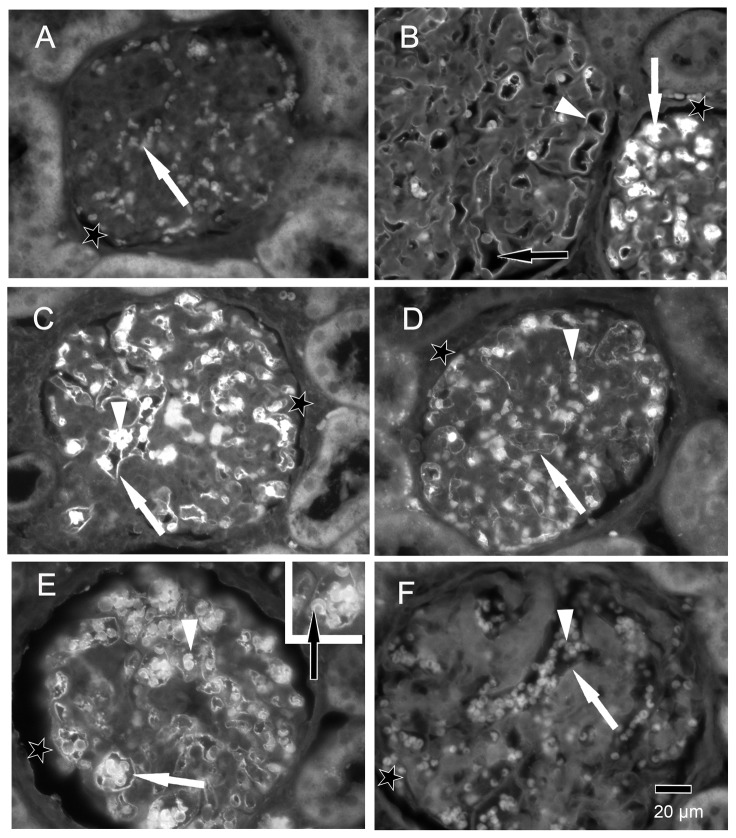
Immune fluorescent photomicrographs of glomeruli from control (A), aflibercept-treated (B–D) and ranibizumab-treated (E–F) monkeys eyes. In all figures, the asterisks label the spaces of the Bowman capsule. **A**) Kidney sections from the control animal did not show any specific staining with anti-human IgG-Fc antibody in the glomeruli. Only the erythrocytes (arrow) within the capillaries showed a weak fluorescence. **B**) One day after aflibercept injection, the endothelium cell layer and material within the capillaries of a glomerulus were highly fluorescent (white arrow) after labelling with an antibody against the Fc region of IgG. In an adjacent glomerulus, only the endothelium was stained (white arrowhead) whereas the lumina of the vessels did not contain IgG-Fc positive material (black arrow). **C**) Erythrocytes within the glomeruli (arrowhead) as well as the endothelium (arrow) were highly fluorescent. **D**) Seven days after aflibercept injection, the fluorescent material within the capillaries (arrowhead) and the fluorescence intensity of the endothelium became weaker. **E**) One day after ranibizumab injection, the endothelium cell layer (white arrow) and erythrocytes (arrowhead and black arrow in the inset) were fluorescent after staining with an antibody against human Fab of IgG. **F**) The specific fluorescence of the endothelium (arrow) and erythrocytes (arrowhead) was nearly lost seven days after injection of ranibizumab.

Kidney sections from the control animal did not show any specific staining in the glomeruli after staining either with an antibody against the anti-human IgG-Fc fragment (Fig. 4A) or against the anti-human Fab fragment of IgG (not shown). Only the erythrocytes within the capillaries showed a weak autofluorescence (Fig. 4A). After omitting the first antibodies, the same staining pattern as in Fig. 4A was observed (not shown). One day after aflibercept injection, the endothelium cell layer and material within the capillaries of many glomeruli were highly fluorescent (Fig. 4B–C). Occasionally, glomeruli in which only the endothelium was stained were localised close to others that contained high amounts of IgG-Fc reactive material within the capillaries (Fig. 4B). Erythrocytes within the glomeruli were highly fluorescent (Fig. 4C). Seven days after aflibercept injection, the fluorescent material within the capillaries was fewer and the fluorescence intensity became weaker (Fig. 4D). One day after ranibizumab injection, the endothelium cell layer and erythrocytes of most glomeruli became fluorescent after labelling with an antibody against the human Fab fragment of IgG (Fig. 4E). However, the specific fluorescence was nearly lost seven days after injection of ranibizumab (Fig. 4F).

#### **1.2 Quantification and normalisation of ranibizumab/aflibercept stainings.**

**Figure 5:**
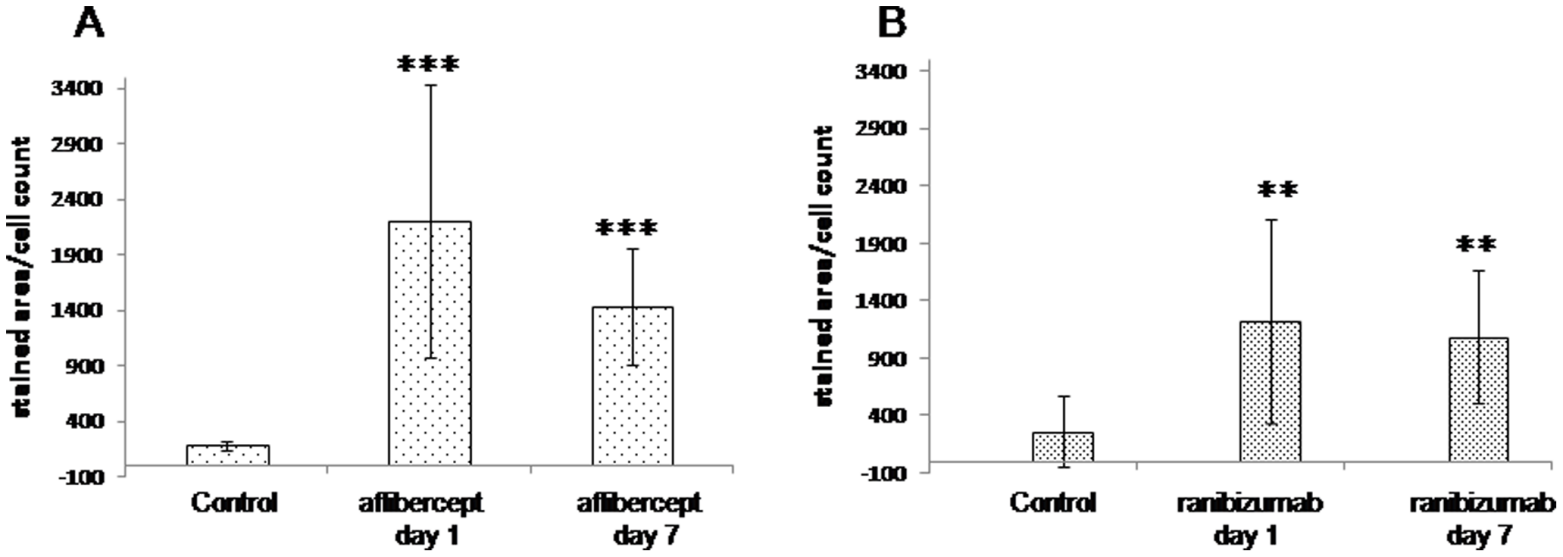
Quantification and normalisation of aflibercept/ranibizumab staining. Results of the analysis of aoi of glomeruli from kidneys of monkeys one and seven days after aflibercept (A) or ranibizumab (B) treatment and the corresponding controls, respectively after staining with the anti-Fc-fragment antibody for aflibercept and the anti-Fab fragment antibody for ranibizumab immune reactivity analysis; t-test against the corresponding control was performed: ** for p<0.001,*** for p<0.0001.

Immune reactivity against aflibercept or ranibizumab was only detected in aflibercept- or ranibizumab-treated animals respectively and did not show any significant differences between days one and seven (Fig. 5A–B).

#### **1.3 Quantification and normalisation of VEGF staining.**

**Figure 6:**
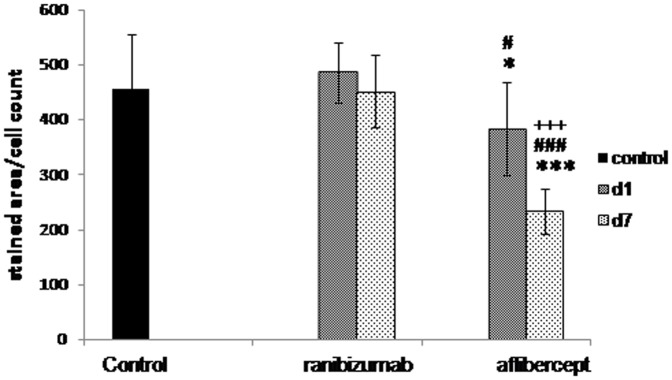
Quantification and normalisation of the VEGF staining. Results of the analysis of aoi of glomeruli from kidneys of monkeys one and seven days after ranibizumab and aflibercept treatment and the corresponding controls after anti-VEGF staining; t-test against control: * for p<0.05, *** for p<0.0001; t-test ranibizumab day 1 *versus* aflibercept day 1 and ranibizumab day 7 *versus* aflibercept day 7: # for p<0.05, ### for p<0,0001; t-test aflibercept day 1 *versus* aflibercept day7: +++ for p<0.0001.

The anti-VEGF stained area to cells ratio in the aoi of the samples (representing the mean VEGF level/cell ratio) from the tissue of the ranibizumab-treated animals showed no significant changes at any of the analysed time points (486±55 (day 1) and 451±66 (day 7)) compared to the untreated control-animal samples (456±99). However, the stained area to cells ratio of the tissue-samples of the aflibercept-treated animals showed a significant decrease one day (383±85) after treatment (p<0.05) and an even stronger decrease seven days (233±41) after treatment (p<0.0001) compared to the untreated control-animal samples (456±99). The levels of VEGF were found to be significantly lower in the aflibercept-treated animals on days one (383±85 vs. 486±55; p<0.05) and seven (233±41 vs. 451±66; p<0.0001) after treatment as compared to the corresponding ranibizumab-treated ones. The decrease of the VEGF level from day one to day seven after treatment was also found to be significant (383±85 vs. 233±41; p<0.0001) (Fig. 6).

### 2. Measurement of VEGF-A^165^ plasma levels

All doses were below the detectable limit of the assay meaning under 30 pg/ml (not shown).

### 3. General examinations by light and transmission electron microscopy

The biopsies of all the kidneys were first studied by light microscopy and also by TEM with increasing magnification in order to scan the specimens on artefacts and pathologic features. None of the glomeruli was sclerotic. The podocyte foot processes were well-formed and did not show effacement, and a continuous slit diaphragma could be observed. The glomerular basement membrane (GBM) was of normal thickness and did not show duplication in all specimens without widening of the subendothelial space or cellular interposition. The well-defined glomerular endothelium was flattened though there were slight variations in thickness without endotheliosis. In all samples, it was mostly adjacent to the GBM.

**Figure 7:**
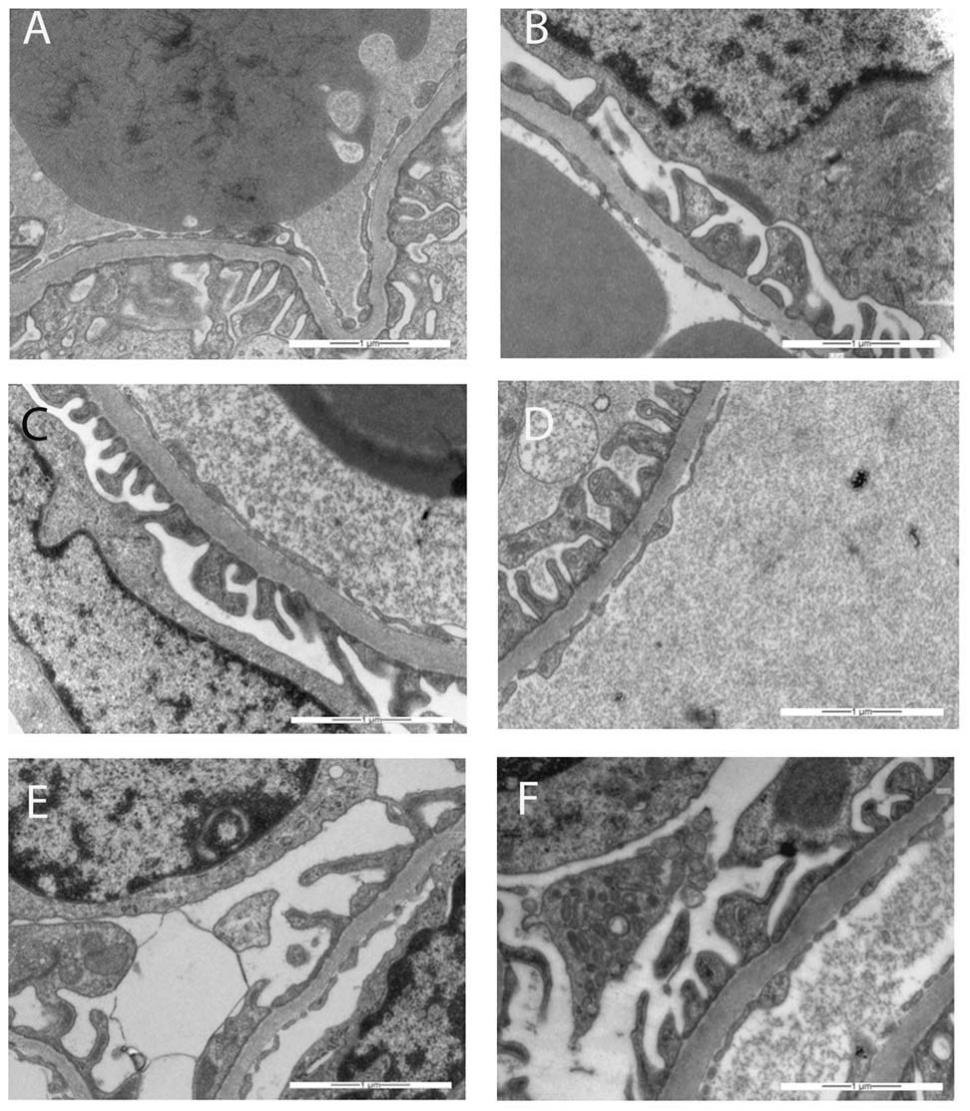
Examples of transmission electron micrographs used for the quantification of the glomerular endothelial cells fenestrations. The red line drawn a long lamina rara interna, the length of line is in µm, the red crosses point out fenestrations. (**A**) after injection of the vehicle; (**B**) in the untreated control; (**C**) one day after injection of ranibizumab; (**D**) seven days after injection of ranibizumab; (**E**) one day after injection of aflibercept; (**F**) seven days after injection of aflibercept. Magnification ×20000.

The capillary loops were filled with more or less electron-dense serum. The density difference could be observed in glomeruli of all of the kidneys (Fig. 7).

### 4. Quantification of the glomerular endothelial cells fenestrations

**Figure 8:**
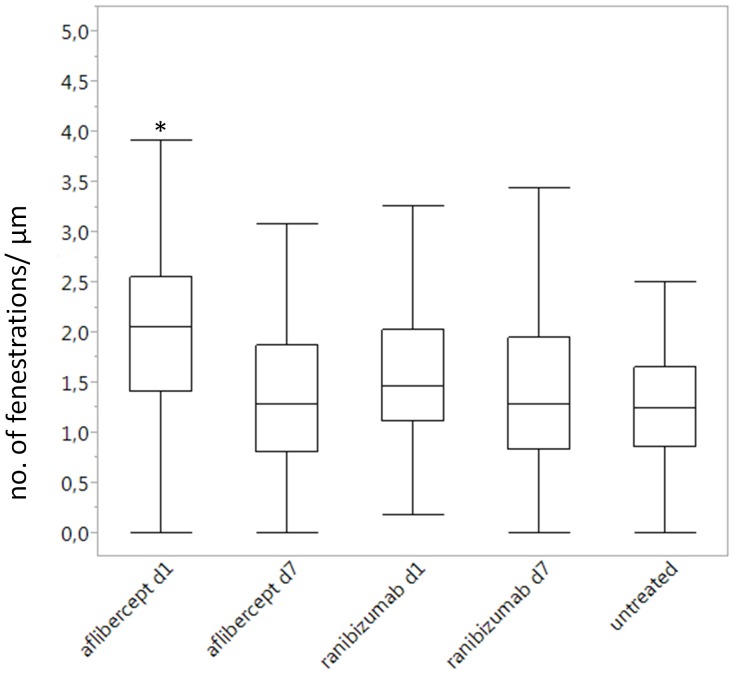
Box plot representation of the quantification of the fenestrations per µm depending on the treatment and its duration. Application of the Student's test showed no significant differences in the number of fenestrations per µm except one day after aflibercept's injection where the number was increased compared to all other groups (*, p<0.05).

In total, 30 glomeruli e.g. three glomeruli per monkey fulfilling the criteria described above were investigated. The total amount of pictures taken was 4855 of which 1327 (27.3%) were evaluated. After the ratio of fenestrae per µm was calculated for each image, the values obtained for the two monkeys of aflibercept and ranibizumab for each time point were pooled. Student's test was performed with values of the untreated monkey as control values and it showed a statistically significant increase (p<0.05) in the number of fenestrae per µm one day after aflibercept injection (median: 2.05) compared to all other groups (medians: 1.27 for aflibercept day 7, 1.46 for ranibizumab day 1, 1.29 for ranibizumab day 7 and 1.25 for the untreated group) (Fig. 8). The other conditions did not show any statistical significance compared to the controls (Fig. 8).

## Discussion

In the normal kidney, VEGF is expressed on podocytes. Dysregulation of VEGF expression within the glomeruli has been associated with a wide range of renal diseases that may occur within weeks to months after intravitreal administration of VEGF inhibitors [14], [27]. The dose of anti-VEGF agents used in ophthalmology is minute compared with that used intravenously, but there is evidence of systemic absorption and diffuse inhibition of VEGF [14], [17], [38], [39]. Pelle *et al*., reported a non diabetic patient with normal kidney function who developed kidney toxicity after four injections of ranibizumab for the treatment of age-related macular degeneration [14]. Several studies evaluating the safety and efficacy of intravitreal anti-VEGF in diabetic patients have reported renal adverse effects [17], [39], [40]. Georgalas et al., postulated that plasma levels of anti-VEGF could possibly cause deregulation of VEGF expression in the kidney and cause renal damage. Of course the fact that the patients were diabetic should be taken into consideration since such patients are prone to develop renal failure [17]. Previous studies [41] have shown that intravitreally injected ranibizumab is cleared from the eye into the circulation with a half-life of approximately three days in monkeys [42] and that ranibizumab can be found in the serum but the concentrations detected were considered below the necessary threshold for a sufficient VEGF inhibition [43]. Since ranibizumab is not protected from serum elimination by an Fc-fragment, it has been suggested that it is rapidly cleared from the circulation *via* renal elimination. Our data is in accordance with these studies since indeed ranibizumab was indeed localised in the glomeruli one and seven days after intravitreal injection and it did not affect the VEGF level in the glomeruli nor the number of glomerular endothelial fenestrations. We were unable to measure plasma VEGF levels since the concentrations were always below the detectable limit (30 pg/ml) of the assay. Larsson et al., have determined in 80 plasma samples of healthy humans that the mean value ± SD of VEGF was 32±21 pg/ml [44]. Moreover, the use of serum instead of plasma would be preferable since serum VEGF levels are several fold higher than plasma levels, because platelets express VEGF and secrete it during blood clotting [45].

Unfortunately we have no data concerning the urinary sediments. It would be of great interest to analyse them, especially as there are indications that anti VEGF therapies have influence on the kidneys, and can cause proteinuria and hypertension [3]–[5]. The analysis of the urinary sediments will also be an aim of our further studies.

It has been published that aflibercept, in contrast to bevacizumab, binds VEGF-A in a 1∶1 stoichiometry which remains stable in the circulation [46]. The authors speculated that because of this stoichiometry and the inert nature of the complexes, aflibercept is not expected to accumulate in renal glomeruli as has been found for bevacizumab [46]. The present study shows for the first time that in reality aflibercept is found in the glomeruli after intravitreal injection and moreover that it significantly decreases their VEGF level. Whether pathological alterations were caused by ranibizumab or aflibercept in a different manner or not is out of the focus of this pilot study. Surprisingly one day after injection, aflibercept was able to increase the number of glomerular endothelial cells fenestrations compared to the controls or ranibizumab-treated monkeys. Since the glomerular VEGF level was reduced, we rather expected a reduction or a stabilisation of the number of fenestrations, as apparent in the choriocapillaris after intravitreal injection of an anti-VEGF agent [19]. This result suggests a complex regulation of glomerular endothelial cell fenestrations which is not completely elucidated. Indeed in contrast to the choriocapillaris, it has been shown that the fenestration of glomerular capillaries requires the action of TGF-β1 [47]. Moreover, in systemic endothelial fenestrations, the intracellular pathways through which VEGF acts to induce fenestrations include a key role for the fenestral diaphragm protein plasmalemmal vesicle-associated protein-1 (PV-1). However, the role of PV-1 in glomerular endothelial cell fenestrations is less clear, not least because of controversy over the existence of glomerular endothelial cell fenestral diaphragms [48]. Satchell and Braet thought that the glomerular endothelial cell fenestrations generally do not express PV-1 and, it is generally asserted, do not possess diaphragms. However, they should note that a number of observations challenge this position. To some extent, appearance of this feature may be dependent on the fixation and labelling techniques used, since a diaphragm is seen in some preparations but not in others. It could be either that some techniques destroy a very delicate diaphragm or that other techniques result in an artefactual appearance of a diaphragm, perhaps through condensation/cross-linking and labelling of glycocalyx, other plasma proteins, or the outer surface of the glomerular basement membrane. Another careful study demonstrated that glomerular endothelial cells in the rat adult kidney, apart from a small fraction, do not furnish diaphragms with their fenestrae; most glomerular endothelial cells in the immature glomeruli of rat embryos have diaphragmed fenestrations and the number of glomerular endothelial cells with diaphragmed fenestrations is increased in the glomeruli of Thy-1.1 nephritis rats, presumably reflecting a process of restorative remodelling of the glomerular capillary tuft after injury [49]. Along with the appreciation that the intraglomerular portion of efferent arterioles and direct tributaries may express fenestrated diaphragm [50], this goes a long way toward clarifying the position. In our study, the fenestrations of the glomerular endothelial cells of adult monkeys were clearly not closed by diaphragms (Fig. 3A) which contrast to those observed in the choriocapillaris and reported in one of our previous monkey studies (Fig. 6 in [19]). Since the same technique was used in these two studies, technical artefacts can be excluded. The glomerular changes observed in our study are not very extensive, but one has to keep in mind, that in the present model anti-VEGF treatment was given only once and the animals used have no overt renal pathology. Usually patients with e.g. diabetic retinopathy get the anti-VEGF treatment on regular basis and as proliferative diabetic retinopathy is a microvascular disesase, we have to assume in these patients also microvascular disturbances in the kidney including microalbuminuria. Under this condition VEGFA secreted from podocytes is essential to maintain proper cellular functions [51].

What might happen if VEGFA is affected in a stimulated system is speculative and should be investigated. This might be a consequence of different antibody design. The immune complexes might be inductor of thrombotic microangiopathy in the kidney [52]. Caution is needed when patients which might also have already renal disease get these substances locally, which have definitive systemic effects.

## Conclusions

In conclusion, we showed that intravitreal ranibizumab and aflibercept can escape from the blood-retinal barrier and are also distributed to distant organ like the kidneys. Our study demonstrated that a single dose of intravitreally injected aflibercept already decreases the VEGF level in the glomeruli one and seven days after treatment whereas ranibizumab did not affect the glomerular VEGF level. In clinical practice, it is therefore important to monitor patients receiving intravitreal injection of VEGF inhibitors for possible systemic side-effects, particularly kidney injury, which may not be immediately apparent. Because of the increasing use of intravitreal anti-VEGF agents in the treatment of age-related macular degeneration, as well as for other indications such as diabetic retinopathy, further studies are highly needed to elucidate the effects of repeated anti-VEGF injections on VEGF concentrations in distant organs.

## Supporting Information

Figure S1 **Demonstration of the systematic uniform random sampling protocol (SURS) on a multiple image arrangement (MIA).** Transmission electron microscopy, magnification ×3000. Probe: Aflibercept day 1, Glomerulus 1. Asterisk marks starting position (first picture), double asterisks mark end position (last picture), arrows mark direction into which SURS was performed.
(DOCX)
