# Supplementary figures and images for: Effects of a Single Intravitreal Injection of Aflibercept and Ranibizumab on Glomeruli of Monkeys
Source: PLoS One. 2014 Nov 21;9(11):e113701. doi: 10.1371/journal.pone.0113701 (PMC4240650; doi:10.1371/journal.pone.0113701)

*
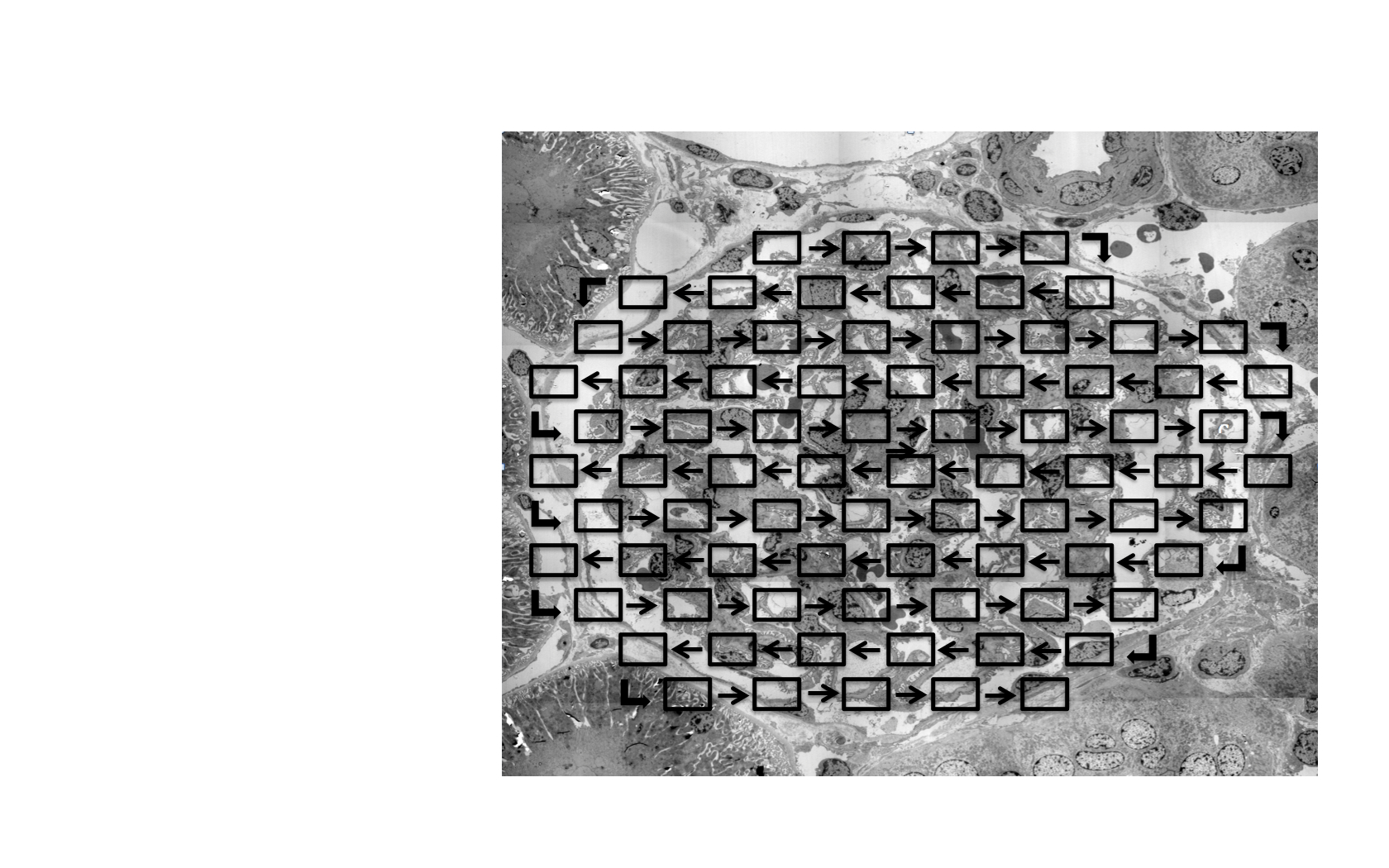
*

Supplement: Figure S1 — Demonstration of the systematic uniform random sampling protocol (SURS) on a multiple image arrangement (MIA). Transmission electron microscopy, magnification ×3000. Probe: Aflibercept day 1, Glomerulus 1. Asterisk marks starting position (first picture), double asterisks mark end position (last picture), arrows mark direction into which SURS was performed. (DOCX) [file pone.0113701.s001.docx]
